# Physiological frailty index (PFI): quantitative in-life estimate of individual biological age in mice

**DOI:** 10.18632/aging.101206

**Published:** 2017-03-19

**Authors:** Marina P. Antoch, Michelle Wrobel, Karen K. Kuropatwinski, Ilya Gitlin, Katerina I. Leonova, Ilia Toshkov, Anatoli S. Gleiberman, Alan D. Hutson, Olga B. Chernova, Andrei V. Gudkov

**Affiliations:** ^1^ Department of Pharmacology and Therapeutics, Roswell Park Cancer Institute, Buffalo, NY 14263, USA; ^2^ Everon Biosciences, Inc., Buffalo, NY 14203, USA; ^3^ Department of Cell Stress Biology, Roswell Park Cancer Institute, Buffalo, NY 14263, USA; ^4^ Biostatistics and Bioinformatics, Roswell Park Cancer Institute, Buffalo, NY 14263, USA

**Keywords:** aging, high fat diet, obesity, rapamycin, rapatar, chronological age

## Abstract

The development of healthspan-extending pharmaceuticals requires quantitative estimation of age-related progressive physiological decline. In humans, individual health status can be quantitatively assessed by means of a *frailty index* (FI), a parameter which reflects the scale of accumulation of age-related deficits. However, adaptation of this methodology to animal models is a challenging task since it includes multiple subjective parameters. Here we report a development of a quantitative non-invasive procedure to estimate biological age of an individual animal by creating *physiological frailty index* (PFI). We demonstrated the dynamics of PFI increase during chronological aging of male and female NIH Swiss mice. We also demonstrated acceleration of growth of PFI in animals placed on a high fat diet, reflecting aging acceleration by obesity and provide a tool for its quantitative assessment. Additionally, we showed that PFI could reveal anti-aging effect of mTOR inhibitor rapatar (bioavailable formulation of rapamycin) prior to registration of its effects on longevity. PFI revealed substantial sex-related differences in normal chronological aging and in the efficacy of detrimental (high fat diet) or beneficial (rapatar) aging modulatory factors. Together, these data introduce PFI as a reliable, non-invasive, quantitative tool suitable for testing potential anti-aging pharmaceuticals in pre-clinical studies.

## INTRODUCTION

Mammalian aging is characterized by a gradual decline of numerous health parameters with multiple biochemical, physiological and behavioral manifesttations [1]. These include reduced muscle strength, bone degeneration, osteoporosis, an increase in systemic inflammation, vascular calcification, hair loss, cataracts, cognitive decline, etc. [2]. Several animal models including *C.elegans*, yeast, *Drosophila* and rodents (rats and mice) have been successfully used over the last several decades to address mechanistic aspects of aging and development of age-related diseases. In most of these studies the major metric parameter for assessing pro-/anti-aging effect of genetic, nutritional or pharmacological manipulation has been the animals' lifespan.

While being informative, longevity by itself however, cannot provide an assessment of the animal's health status, which, like in humans, can significantly decline at older ages and therefore reduce the quality of life. This concern is particularly relevant to research focused on developing the “healthspan”-extending pharmaceuticals, efficacy of which may not be necessarily translated in increased longevity but rather in prolongation of healthy life and require quantitative objective assessment. Clinical studies in humans measure age-related declines in performance by quantifying the frailty index (FI), which reflects accumulation of health deficits during chronological aging [3].

Since numerous studies have shown that many age-associated changes that occur in humans are also present in aged mice, FI was recently introduced as a measure of mouse aging to pre-clinical models [4]. However, standardized and comprehensive approaches for FI measurements, which will address changes in a broad spectrum of physiological functions, are still missing. Here we describe development of an alternative scoring system, based on a selected set of non-invasive quantitative and physiological parameters, which could be repetitively used in the same animal over the course of its entire lifespan. We refer to this set of parameters as physiological frailty index (PFI). We show that similar to results of human studies older mice have much greater PFI reflecting age-related accumulation of deficits. We also validated our approach of PFI by testing detrimental (feeding high fat diet, HFD) and beneficial (treatment with mTOR inhibitor rapamycin) factors on animals' longevity and healthspan and revealed significant sex-dependent differences, thus emphasizing the importance of including both male and female animals in pre-clinical studies. The results of our study provide a feasible tool applicable for pre-clinical studies to test the efficacy of anti-aging pharmaceuticals.

## RESULTS

### Choosing informative parameters for creating PFI

To select which physiological parameters to choose for creating reliable FI we used several criteria. First, we wanted them to be diverse to reflect the status of different health-related physiological systems. Second, we wanted them to be objective and quantitative and not to involve any visual scoring. Finally, we wanted them to be minimally invasive so they can be applied in longitudinal studies. Based on these considerations, we selected 29 variables reflective of physical fitness (body weight and grip strength), cardiovascular system (systolic, diastolic and mean blood pressure, heart rate, tail blood flow and tail blood volume), total blood cell composition (white and red blood cell counts and differentials), plasma concentration of CXCL1/KC, triglycerides and glucose. All variables were measured in male and female NIH Swiss mice of different chronological ages (CA) of 26, 52, 78 and 104 weeks in a cross-sectional study. Next, using one-way ANOVA analysis, we identified those variables that demonstrate statistically significant changes during aging (p-value <0.05). Out of total of 29 parameters, 16 and 18 qualified these criteria in males and females respectively. These parameters and corresponding mean values for each age group are summarized in Tables 1 and 2. Interestingly, the two lists have only partial overlap pointing to sex-specific differences in the process of aging.

Table 1Physiological parameters used to create PFI in male NIH Swiss mice. Data is presented as mean +SEM

**Table:** 

Age, weeks	GS, g	Dia	Mean	Flow	NE (K/μl)	LY (K/μl)	MO (K/μl)	KC (pg/ml)
26	76.96±3.42	79.28±3.98	88.93±3.49	15.81±2.17	1.45±0.12	3.24±0.30	0.19±0.02	595.9±57.22
52	72.17±3.42	76.95±2.24	85.63±2.17	22.07±1.56	1.02±0.17	2.52±0.30	0.217±0.02	905.07±234.12
78	78.74±4.14	72.70±4.16	84.17+4.36	15.86±1.7	2.65±0.56	4.32±0.77	0.309±0.10	372.10±39.58
104	49.92±3.15	88.75±3.33	97.81±3.34	21.04±1.6	0.55±0.14	2.25±0.82	0.271±0.08	705.93±94.94

**Table:** 

Age, weeks	NE %	MO %	RBC (M/μl)	HB (g/dl)	HCT %	MCV(fl)	MCH (Pg)	MCHC (g/dl)
26	24.16±4.04	3.02±0.30	8.91±0.41	13.09±0.69	39.60±2.20	44.27±0.72	14.66±0.23	32.90±0.29
52	26.01±2.09	6.03±0.57	7.97±0.46	11.83±0.73	37.85±2.37	47.34±0.69	14.81±0.21	31.28±0.30
78	34.18±2.12	3.83±0.48	9.94±0.59	15.23±0.87	49.20±2.60	49.71±0.98	15.33±0.22	30.91±0.27
104	18.10±3.16	8.78±1.41	6.6±0.63	9.50±0.97	34.01±3.23	51.73±1.00	14.33±0.0.16	27.77±0.45

GS – grip strength; Dia – diastolic pressure; Mean – mean pressure; NE – neutrophils; LY – lymphocytes; MO – monocytes; RBC – red blood cells count; HB – hemoglobin; HCT – hematocrit; MCV – mean corpuscular volume; MCH – mean corpuscular hemoglobin; MCHC – mean corpuscular hemoglobin concentration

Table 2Physiological parameters used to create PFI in female NIH Swiss mice. Data is presented as mean +SEM

**Table:** 

Age, weeks	BW, g	GS, g	Dia	Flow	WBC (K/μl)	NE (K/μl)	NE %	LY %	MO %
26	26.61±0.31	76.96±3.42	79.28±3.98	15.81±2.17	5.19±0.27	0.86±0.07	16.56±0.96	77.82±0.81	4.90±0.37
52	32.20±1.15	72.17±3.42	76.95±2.24	22.07±1.56	4.41±0.46	1.28±0.14	30.50±4.22	61.90±4.27	7.06±0.66
78	37.17±1.86	78.74±4.14	72.70±4.16	15.86±1.7	7.47±0.91	2.57±0.42	34.23±3.36	59.59±3.90	4.01±0.29
104	35.71±1.17	49.92±3.15	88.75±3.33	21.04±1.6	6.55±2.32	1.57±0.50	22.86±3.69	63.99±4.12	7.90±0.83

**Table:** 

Age, weeks	EO%	RBC (M/μl)	HB (g/dl)	HCT %	MCV(fl)	MCH (Pg)	MCHC (g/dl)	PLT	MPV
26	0.11±0.02	9.86±0.25	14.95±0.19	49.05±2.13	49.47±1.34	14.72±0.15	30.15±0.86	1134.53±28.53	4.83±0.04
52	0.50±0.1	11.21±0.27	16.33±0.31	61.13±1.43	54.65±1.16	14.60±0.31	26.75±0.37	706.00±97.92	4.76±0.11
78	1.70±0.58	10.11±0.42	15.39±0.76	49.12±2.45	48.44±0.77	15.16±0.20	31.35±0.19	1285.09±134.26	5.26±0.09
104	3.95±0.67	9.63±1.07	9.50±0.97	33.49±3.65	48.15±1.05	13.83±0.30	28.72±0.22	876.25±96.58	5.57±0.19

WBC – white blood cells, NE – neutrophils; LY – lymphocytes; MO – monocytes; EO – eosinophils; RBC – red blood cells count; HB – hemoglobin; HCT – hematocrit; MCV – mean corpuscular volume; MCH – mean corpuscular hemoglobin; MCHC – mean corpuscular hemoglobin concentration; PLT – platelets; MPV – mean platelet volume

### Quantification of PFI in chronologically aged male and female NIH/Swiss mice

**Figure 1:**
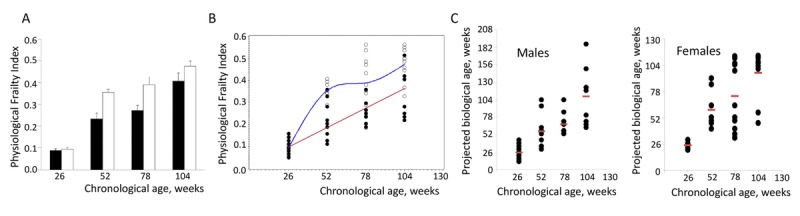
Assessment of individual biological age of NIH Swiss mice (**A**) Age-related increase in PFI in male (closed bars) and female (open bars) NIH Swiss mice (n=10-12/group). PFI indices were measured as described using 16 or 18 parameters for males and females respectively. Data is presented as mean ±SEM. One-way ANOVA detects significant effect of age on FI value (p<0.001 for both sexes). (**B**) PFI values for individual male (closed circles) and female (open circles) mice. A cubic regression performed on this set of data generated the best fitting model as: PFI=0.00684+0.0034×BA for males (red line) and PFI=-0.67372+0.04277×CA-0.00057899×CA^2^+0.00000263×CA^3^ for females (blue line). All regression coefficients presented were significantly different from 0 at the 0.05 alpha-level. (**C**) Projected biological age of individual mice calculated from the PFI values using the fitting model predictions.

To quantitatively evaluate age-dependent accumulation of health deficits, male and female NIH Swiss mice were allowed to age in the animal facility under normal husbandry conditions. Separate groups of male and female mice were tested for their overall health status at the ages of 26, 52, 78, and 104 weeks as described in Materials and Methods section. PFI was created using the 26-week old mice as a reference so that deviation of each parameter from the mean value was calculated. As shown in Fig. 1A, PFI demonstrates gradual increase with age in both males and females; however, dynamics of this process was different. Consistent with previous studies, both in mice and humans [5], females show a more rapid accumulation of health deficits than males and average PFI values were higher compared to the males of the corresponding age.

Although average value of PFI showed highly statistically significant increase with age, within each age-matched group it demonstrated considerable individual variability (Fig. 1B). This is consistent with widely accepted concept originated from human and animal model studies stating that chronological age (CA, the actual age from the date of birth) and biological age (BA, reflecting individual's health status) are different [6-8]. To test whether PFI value can be used as a predictor of an individual animal's BA, polynomial regression of order 3 was fit to the data using a stepwise regression approach.

The best fitting models were given as:

PFI=0.00684+0.0034×BA for males and

FI=-0.67372+0.04277×CA-0.00057899×CA^2^

+0.00000263×CA3 for females.

Next, we used these equations to calculate BA of each individual animal based on its PFI. Fig. 1C presents results of this analysis showing high degree of variability of estimated BA within the group of chronologically age-matched male and female mice. Overall these data indicate that our scoring system allows for an unbiased and reliable quantitation of age-related accumulation of deficits. They also suggest that PFI may be used as a tool for evaluating health status of an individual animal and determining its BA. This tool may be applied for quantitative assessment of the effects of various environmental, nutritional and pharmacological interventions on healthspan.

### Sex-dependent effect of high fat diet on PFI and longevity

To validate our approach for assessing animal's BA, we subjected them to two treatments known to cause detrimental (feeding high fat diet, HFD) or beneficial (administration of rapamycin) effects on health and longevity. Fifty two week old male and female NIH Swiss mice were randomly assigned to four groups. Group 1 remained on regular chow and drinking water; group 2 remained on regular chow but started receiving rapamycin in drinking water. Group 3 started receiving HFD in combination with normal drinking water and group 4 was fed HFD in combination with rapamycin.

**Figure 2:**
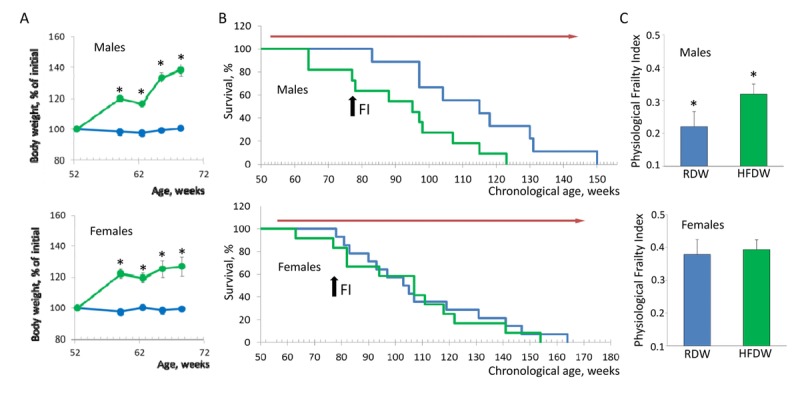
Sex-specific effects of HFD on lifespan and health of NIH Swiss mice (**A**) HFD-induced body weight gain in male and female mice. Feeding HFD results in 40% and 30% increase in body weight in males and females respectively (p<0.001, Student's t-test). Since mortality is usually preceded by rapid weight loss, data is shown for the initial period of treatment before the first case of death in each group was recorded. Blue line – regular diet; green line – HFD. (**B**) Feeding HFD reduces lifespan of male mice from 121.1±9.2 to 91.5±5.9 weeks (p=0.008, Kaplan-Meier log-rank test) but had no effect on longevity of female mice (109.6 ±6.9 and 104.9±7.7 weeks for mice fed regular or high fat chow respectively). Blue line – regular diet; green – HFD. Red arrow indicates the period of time when mice received HFD. Black arrow indicates time when PFI was measured (at the age of 78 weeks). (**C**) PFI created at 78 weeks of age using 16 or 18 parameters for male and female mice respectively. Feeding HFD significantly increases PFI of male (p=0.019, Student's t-test) but not female mice. RDW - regular diet in combination with water (group 1), HFDW – HFD in combination with water (group 3).

It is well established that HFD-induced obesity has a detrimental effect on health increasing the risk of diabetes, heart disease, high blood pressure and cancer [9]. As shown in Fig. 2A, feeding HFD results in significant increase in body weight in both males and females (40% and 30% for males and females respectively). Nevertheless, effect on longevity was very different. Whereas feeding HFD reduced lifespan of male NIH Swiss mice from 121.1±9.2 to 91.5±5.9 weeks (p=0.008, Kaplan-Meier log-rank test), it had no effect on longevity of female mice (109.6 ±6.9 and 104.9±7.7 weeks for mice fed regular or high fat chow respectively; Fig. 2B). This result is consistent with previous studies showing that female mice are protected against HFD-induced metabolic changes by maintaining an anti-inflammatory environment in the intraabdominal adipose tissue, whereas HFD-fed male mice develop adipose tissue inflammation, glucose intolerance, hyperinsulinemia, and islet hypertrophy [10].

To further test whether reduction in lifespan in males correlates with an increase in accumulation of health deficits, after 26 weeks of feeding HFD (at the age of 78 weeks) we created PFI for each individual animal. Consistent with longevity data, feeding HFD significantly increased PFI in male but not in female mice (Fig. 2C). These data indicate that HFD-induced obesity produces stronger damaging effect in male mice affecting both their longevity and health status and that our approach can reliably detect overall health decline in a quantitative manner.

### Chronic treatment with rapamycin increases the lifespan of female NIH Swiss mice without affecting their health status

**Figure 3:**
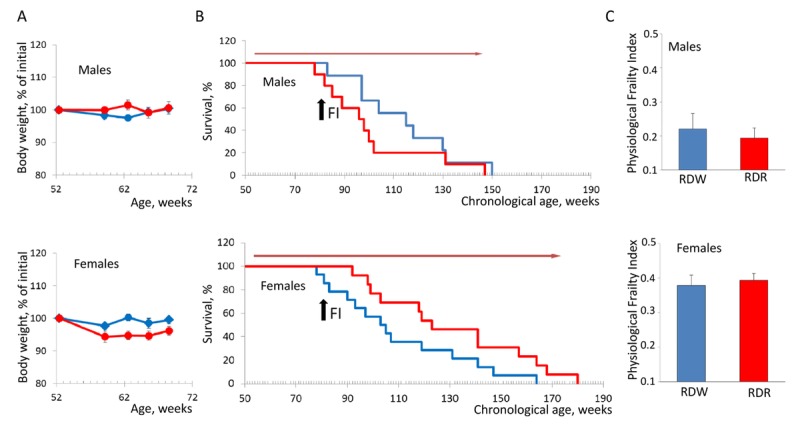
Sex-specific effects of rapamycin on lifespan and health of NIH Swiss mice (**A**) Animals receiving rapamycin in drinking water maintain their body weights comparable to control mice. Blue line – normal drinking water; red line – water with rapamycin. (**B**) Kaplan-Meier survival curves for mice fed regular chow in combination with normal drinking water (blue line) or rapamycin (red line). Chronic administration of rapamycin has no effect on longevity of male mice (mean survival is 113.91±6.98 and 100.8±6.96 weeks for control and rapamycin-treated mice respectively. In females, rapamycin administration increases lifespan from 110.09±7.12 to 131.23±8.29 weeks (p=0.05, Kaplan-Meier log-rank test). Red arrow indicates the period of time when mice received rapamycin. Black arrow indicates time when PFI was measured. (**C**) PFI created at described above. No effect of rapamycin on health status was detected in male and female mice fed regular chow. RDW – regular diet in combination with normal drinking water (group1); RDR – regular diet in combination with rapamycin (group 2).

To further validate our method, we decided to test whether we can reliably measure the beneficial effects of lifespan increasing approaches on animals' health. For this we chose to use rapamycin, which has been shown to extend lifespan of inbred and genetically heterogeneous mice [11, 12] as well as of mice that develop syndrome of premature aging [13]. Several studies performed in cancer-prone animal models suggest that rapamycin extends lifespan by delaying tumor development and progression [11, 14, 15]. In our previous work, we successfully used nanoformulated water soluble rapamycin (rapatar), which can be administered with drinking water and demonstrated high bioavailability [14]. In concurrence with the previous study, chronic administration of rapatar did not cause any toxicity as both male and female mice in control and experimental groups maintained similar body weight (Fig. 3A). However, there was a significant difference in the effect of rapamycin on longevity between the sexes. Chronic rapamycin treatment had no effect on longevity of male mice (mean survival 113.91+6.98 and 100.8+6.96 weeks for control and rapamycin-treated mice respectively) whereas in females, it increased lifespan from 110.09+7.12 to 131.23+8.29 weeks (p=0.05, Kaplan-Meier log-rank test; Fig. 3B). Surprisingly, this effect was not translated into improved health status between the groups as monitored by PFI (Fig. 3C). Thus, our data provides an additional support for the concept that chronological and biological aging may not be intrinsically identical and that other factors besides time may affect the pace of age-dependent functional decline.

### Rapamycin alleviates detrimental effect of high fat diet on male mice longevity and healthspan

**Figure 4:**
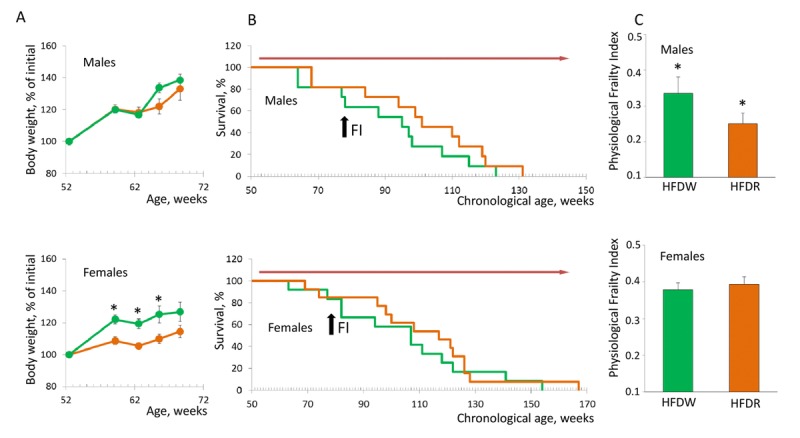
Chronic treatment with rapamycin ameliorates HFD-induced health decline in male mice (**A**) Rapamycin prevents HFD-induced weight gain in female but not in male mice (p<0.01, Student's t-test). Green – HFD given with normal water, orange – HFD given in combination with rapamycin. (**B**) Kaplan-Meier survival curves for mice fed HFD in combination with normal drinking water (green line) or rapamycin (orange line). Chronic administration of rapamycin has no effect on longevity of both male (mean survival is 91.5±5.9 and 100.5±6.26 weeks) and female mice (mean survival is 104.9±7.7 and 110.5±7.6 weeks for control and rapamycin-treated mice respectively). Red arrow indicates the period of time when mice received rapamycin. Black arrow indicates time when PFI was measured. (**C**) PFI created at 78 weeks of age using 16 or 18 parameters for male and female mice respectively. Chronic administration of rapamycin ameliorates detrimental effect of HFD and brings the PFI values down to the normal range characteristic for this age (p=0.014, Student's t-test). HFDW – high-fat diet in combination with regular drinking water (group 3), HFDR – high-fat diet in combination with rapamycin (group 4).

Our data show that HFD-induced obesity shortens lifespan and deteriorates health status of NIH Swiss male mice (Fig. 1). It has been previously demonstrated that in rodents HFD-induced obesity increases activation of mTOR pathway in liver and skeletal muscle [16]. Therefore, we hypothesize that treatment with rapamycin can ameliorate detrimental effects of HFD. Comparison of the dynamics of body weight change showed that chronic treatment with rapamycin did not affect HFD-induced obesity in male mice but did prevent it in females (Fig. 4A). Longevity was not affected in mice of both sexes (Fig. 4B) constituting 91.45±5.87 and 100.55±6.26 weeks for male (p=0.26) and 105.01±7.74 and 110.50±7.58 weeks for female mice (p=0.6). Strikingly, rapamycin administration significantly alleviated detrimental effects of HFD in male mice as judged by a significant decrease in PFI (Fig. 4C). Thus, whereas chronic treatment with rapamycin had no effect on lifespan and health status of male mice under standard diet conditions, it did prevent development of HFD-induced decline in health.

**Figure 5:**
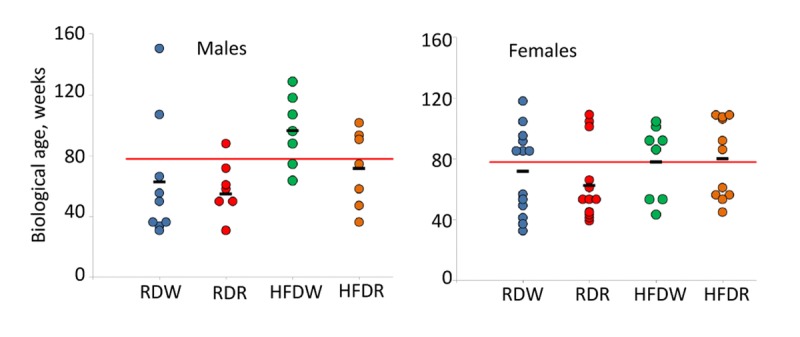
Sex-specific effects of detrimental (HFD) and beneficial (rapamycin) factors on BA of NIH Swiss mice Feeding HFD accelerates aging of NIH Swiss male mice whereas rapamycin counteracts this process. Projected biological age of individual mice (shown by circles) and mean BA for the group (black marker) were calculated from the corresponding FI value using the fitting model predictions. Red line designates chronological age of all mice at the time of testing (78 weeks). Data show that projected BA of all mice that received HFD (green circles) is significantly higher that their actual chronological age and mean BA age for control group on regular diet (62.7±13.3 and 96.4±8.8 weeks for RDW and HFDW groups respectively (p=0.03, Student's t-test). Chronic administration of rapamycin reduces BA of males fed HFD to values characteristic for control group (from 96.4±8.8 to 71.5±9.6 weeks; p=0.04, Student's t-test). No difference between groups was detected in female mice, in which BA was very close to their CA. Slight reduction in BA in rapamycin treated group from 71.8±7.8 to 62.6±7.0 weeks was not statistically significant (p=0.3 Student's t-test).

To further illustrate the complex effects of diet and rapamycin on animal's health, we calculated projected biological age of each 78 week old male and female mouse based on their PFI. Results of this analysis are presented in Fig. 5. Similar to our data for chronologically aged mice, we observed a significant individual variability within each experimental group of age-matched animals. The data showed that feeding HFD increases mean BA of male mice by 34 weeks (from 62.7±13.3 to 96.4±8.8 weeks; p=0.03, Student's t-test; Fig. 5A). Chronic treatment with rapamycin improves animals' health status and reduces their BA to values characteristic for control group (from 96.4±8.8 to 71.5±9.6 weeks (p=0.04, Student's t-test; Fig. 5B). No difference between groups was detected in female mice, in which BA was very close to their CA in all groups. Slight reduction of BA in rapamycin treated group from 71.8±7.8 to 62.6±7.0 weeks was not statistically significant (p=0.3 Student's t-test).

**Figure 6:**
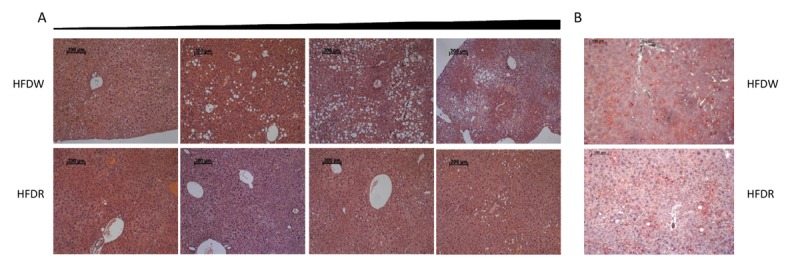
Rapamycin prevents development of HFD-induced hepatic steatosis in male mice (**A**) H&E staining of representative liver sections graded from the least affected (left) to the worst affected (right) within each group. (**B**) Representative oil-red O stained sections of livers. HFDW – mice fed with HFD; HFDR – mice received HFD in combination with rapamycin.

To confirm the preventive role of rapamycin in the development of diet-induced obesity, at completion of the experiment we performed hematoxylin-eosin and Oil Red O staining of livers of male mice fed HFD in combination with either normal water or rapamycin. Fig. 6A shows examples of H&E staining that were graded from the best to the worst within each group demonstrating larger and more lipid droplet accumulation in liver parenchyma in high-fat diet group and significant improvement of hepatic steatosis by rapamycin administration. These conclusions were consistent with the results of the Oil Red O staining (Fig. 6B). Taken together, these data suggest that our approach for frailty assessment allows for reliable and quantitative evaluation of animal's health status both during normal aging process as well as after various interventions.

## DISCUSSION

Aging is characterized by accumulation of deficits and increased frailty, which in turn increases vulnerability of an older organism to various stressors. In clinical gerontology two major tools were developed to evaluate frailty in elderly: the phenotype model and FI (reviewed in [17]). The phenotype model describes frailty as a phenotype that can be scored using 5 criteria of patient's physical performance [18]. In contrast, FI represents cumulative score of a degree of deviation that multiple behavioral, physical and physiological parameters developed with age compared to normal values characteristic to healthy young individuals [3]. Since FI was introduced to human gerontology, it was used in many epidemiological and clinical studies to evaluate the risk of adverse health events in elderly. These include a decline in overall activity, reduced muscle strength, bone degeneration, osteoporosis, an increase in inflammation, vascular calcification, hair loss, cataracts, cognitive decline, etc.

The strategy of estimating FI as a measurement of health status has been also applied to the mouse models [19, 20]. However, the majority of frailty assessment tools use multiple observational approaches such as recording and scoring of visible pathologies (eye inflammation or cataracts, high respiratory rate, visible tumors, alopecia, dermatitis, distended abdomen etc.), or behavioral signs (circling behavior, reduced or excessive grooming, etc.). Although informative, this approach has some significant limitations and researcher bias. First, these parameters are subjective by nature and require scoring by several independent raters in consistent manner [21-23]. Secondly, many health problems start a lot earlier than could be detected by their visual manifestation. Therefore, the goal of our study was to develop a reliable, quantitative scoring system, physiological frailty index (PFI) that would be based on non-invasive quantitative measurements of various physiological parameters, which could be repetitively used in the same animal during the course of its entire lifespan and would represent its overall health status.

Several previous studies suggest that the more diverse variables are used for creating the FI, more reliable FI value is [24]. At the same time to optimize the robustness of the procedure in order to make it applicable to a large number of animals, we were inclined to minimize the number of variables. This rationale was supported by several previous studies, which reported good correlation between FI calculated with 8 and 31 items [24]. Therefore, after measuring 29 diverse physiological parameters including physical (body weight and grip strength), blood cell composition, metabolic, and immune, we selected those that show statistically significant change with age. These parameters were used to create PFI of individual mice of different chronological age. The observed gradual increase in mean PFI values with age suggests that our approach can reliably detect the scale of age-dependent health deterioration in a quantitative manner.

Interestingly, the dynamics of deficit accumulation appeared to be very gender-specific. Thus, in male NIH Swiss mice increase in PFI can be approximated by linear function suggesting that health deficits are evenly accumulated with age. In females the dynamics of increase in PFI is more complicated with sharp increase observed between 26 and 52 weeks of age followed by a period of almost no change (between 52 and 78 weeks) and a second increase after 78 weeks. This is an important observation that has to be considered in both pre-clinical and clinical studies. First, it points to fundamental sex-related differences in the process of aging and consequently, has to be taken into account when developing strategies for treatment of aging and age-related diseases. Second, our data provide an additional explanation for numerous examples of sexual dimorphism in response to life-extending genetic or pharmacological interventions. Although clear mechanistic details of this phenomenon are still not understood, most of the previous reports linked it to interactions of the interventions with sex hormones, sex differences in immune function or in distribution of adipose tissue and accordingly differences in the effects of its secretory activity (reviewed in [25]). Our results suggest that in addition to basic gender differences, females' response to treatments may vary depending on the age at which it is applied. Thus, based on observed sex-related differences in the pattern of deficits accumulation, the lack of effects of both detrimental and beneficial interventions on the health status of female mice may be explained by the fact that treatments started at 52 weeks of age (beginning of “no-change” period in females) and the FI was measured at the end of this phase (78 weeks).

Our results in the experiment with chronic rapamycin treatment showing an increase in lifespan of female NIH Swiss mice without improvement in their health, underscores the importance of using health status as the major metrics in development of anti-aging therapeutics. These data are consistent with previous observations made in different model systems demonstrating that increase in longevity is not necessarily accompanied by improved health status. Such as, caloric restriction extends lifespan in *Drosophila* without improving their cognitive function [26]. Long-lived calorie-restricted mice and IGF-1 knockout mice develop multiple health deficits [27, 28]. Recent work in *C. elegans*, which measured simultaneously worms' longevity and health status, demonstrated that four different long-lived mutants that were used in the study increased the proportion of time spent in a frail state [29]. Altogether, we suggest that our approach allows for unbiased and reliable assessment of healthspan by quantitation of age-related accumulation of deficits during chronological aging than may serve as a valuable tool in a variety of pre-clinical aging studies. Importantly, experimental validation of this approach demonstrated its ability to quantitatively evaluate detrimental effect of HFD on animal's health as well as the ability of rapamycin to mitigate it, thereby providing an alternative age-accelerated model for testing the effects of various environmental, nutritional and pharmacological interventions on healthspan.

## MATERIALS AND METHODS

### Animals

Male and female Cr:NIH(S) Mice (NIH Swiss) mice were purchased from Charles River at the age of 6-8 weeks and were allowed to age at the RPCI Animal Facility. During this time mice were housed 1-3 per cage and were fed ad lib with standard chow (Tekland Global 18% Pretein Rodent Diet). To quantitatively evaluate age-dependent decline in animals' health, FI was created for individual male and female mouse at 26, 52, 78 and 104 weeks of age as described below using cross-sectional experimental design (10-14 mice per group). In a separate experiment, male and female mice (9-14 per group) were randomly assigned to four groups. Group 1 remained on regular chow and drinking water; group 2 remained on regular chow but started receiving rapamycin in drinking water. Group 3 started receiving high fat diet (Harlan Laboratories, TD.03584, 35% Lard Diet) in combination with normal drinking water and group 4 received HFD in combination with rapamycin. Rapamycin was delivered in the form of Rapatar (polymeric formulation developed by Everon Biosciences as previously described [14] at a concentration of 125 mg/L (corresponds to 2.5 mg/L rapamycin). Based on the assumption that mice drink 3-5ml water per day, we estimated that in average each animal received 7.5-12.5 μg rapamycin daily. To preserve rapamycin stability in was delivered in non-acidified water in light-protected water bottles, which were replaced twice/week. When mice were 78 weeks of age FI was created for each individual animal as described below. Mice remained on their designated feeding/water schedules for the rest of the experiments and their longevity was recorded. Data for males and females were analyzed separately. All animal studies were conducted in accordance with the regulations of the Committee on Animal Care and Use at Roswell Park Cancer Institute.

### Grip strength measurement

Fore limb grip strength measurements were performed using Animal Grip Strength System (San Diego Instruments). Five measurements were recorded for each individual animal and the average value was assigned.

### Non-invasive measurement of hemodynamic parameters

Non-invasive measurement of hemodynamic parameters was performed using CODA apparatus (Kent Scientific) according to manufacturer's protocol. Mice were placed into cylinder-shaped restraint devices and allowed to acclimate for 5 min on a heating platform before blood pressure measurements begin. Body temperature was continuously monitored by observation of animal behavior, tail blood volume and an infrared thermometer. Recorded hemodynamic parameters include systolic, diastolic and mean blood pressure, heart rate, tail blood flow and tail blood volume.

### Blood samples collection

To evaluate age-dependent changes in blood composition, blood samples were collected using the least invasive method that does not require anesthesia or restraining. Twenty μl of blood was collected from a single submandibular vein bleed into EDTA-treated Vacutainer tubes (BD) and used for whole blood cell counts and glucose measurements. Another 75ul of blood was collected into Li-Heparin treated plasma separator tubes; plasma was purified by centrifugation at 5000 x g for 5 min and used for measuring concentration of circulating pro-inflammatory cytokines and triglycerides.

### Whole blood cell counts, blood chemistry and inflammatory cytokines

Whole blood cell analysis was performed in 20 μl of blood using Hemavet 950 Analyzer (Drew Scientific). The following parameters were measured: while blood cell counts (WBC), neutrophil (NE), lymphocyte (LY), monocyte (MO) and eosinophil (EO) counts and percentage, red blood cell (RBC) counts, hemoglobin (Hb), hematocrit (HCT), mean corpuscular volume (MCV), mean corpuscular hemoglobin (MCH), mean corpuscular hemoglobin concentration (MCHC), red cell distribution width (RDW), platelet (PLT) counts and mean platelet volume (MPV). Plasma concentration of chemokine (C-X-C motif) ligand 1(CXCL1/KC) was measured using ELISA kit (R&D) according to manufacturer's protocol. Plasma concentration of triglycerides was measured using triglyceride quantification Kit (Abcam) according to manufacturer's protocol. In both assays reactions were run in duplicates and concentrations were calculated from a calibration curve generated for each experiment. Glucose concentration was measured using AlphaTRAK 2 Blood Glucose Monitoring Kit (Abbott Laboratories).

### Creating physiological Frailty Index

Frailty Index was created for each individual mouse as previously described [20] using 26 week-old group as a “young mouse” reference. For each parameter mean value and standard deviation were calculated. Animals differing in more than one standard deviation (STDEV) from mean value in any single parameter were excluded from the reference group. Value for each parameter measured for mice of older ages (52, 78 and 104 weeks) was compared with corresponding value for the reference group and assigned a score. Values that differ less than 1 STDEV were assigned the score of 0 (no deficit, within the range of the reference group). Values that were different for one STDEV were scored as 0.25 (minimal deficit). Values that differ from the corresponding values in the reference group by 2 STDEV were scored as 0.5 and those that differ by 3 STDEV were scored as 0.75. If the value is above 3 STDEV it was scored as 1 (extreme deficit). The number of deficits the individual mouse is expressed was calculated as a ratio of total number of parameters measured and was referred to as Physiological Frailty Index (PFI).

### Histological evaluation

After completion of the experiment mouse livers were fixed in 10% neutral formalin for 24 h. For morphological observations samples were transferred to 70% ethanol and processed in an automated processor (Leica ASP 300) and embedded in paraffin using LEICA EG 1150H embedding unit according to manufacturer's protocols. Five micron sections were obtained using rotary microtome (LEICA RM 2235) and stained with hematoxylin and eosin (H&E). Neutral lipids were revealed by Oil Red O staining according to standard protocol on 12 micron cryo-sections prepared from formalin-fixed material using CM1900 cryostat. Histopathological examination was performed using Zeiss AxioImager A1 with Axiocam MRc digital camera.

### Statistics

Survival curves were generated using Kaplan-Meier estimators and compared using the log-rank test. Continuous data are expressed as mean ± SEM. Statistical analyses were performed using one or two-way ANOVA with Tukey post hoc tests for multiple comparisons, ANOVA on ranks with Dunn's tests or Student's t-test where appropriate. P-values <0.05 were considered significant. To generate a trend line that would best describe age-dependent increase in FI, a cubic regression was fit regressing the PFI on chrono-logical age (CA), CA^2^ and CA^3^. A stepwise selection procedure was then used to eliminate unnecessary terms for any or all of the higher order polynomial terms. The models were fit individually by sex.
